# BioTM Buzz Volume 4, Issue 3

**DOI:** 10.1002/btm2.10144

**Published:** 2019-09-11

**Authors:** Aaron C. Anselmo

**Affiliations:** ^1^ Division of Pharmacoengineering and Molecular, Pharmaceutics, Eshelman School of Pharmacy University of North Carolina at Chapel Hill Chapel Hill North Carolina

## INTRODUCTION TO SPECIAL ISSUE

This special issue of *Bioengineering & Translational Medicine* (Volume 4, Issue 3) is the second issue dedicated to the 2018 ECI Nanotechnology in Medicine conference, held from June 5 to 9 in Albufeira, Portugal. The conference chairs, Dr. Millicent Sullivan of the University of Delaware and Dr. Josué Sznitman of Technion‐Israel Institute of Technology, served as Guest Editors for both of these special issues. As a reminder, the key thematic focus area at the 2018 meeting was “bridging translational in vitro and in vivo interfaces” and the conference‐highlighted challenges associated with design and integration of platforms to evaluate nanotechnology performance. This second special issue features articles that address these challenges. Below, we highlight two of these articles.

## CONSIDERING VASCULAR HETEROGENEITIES

Navigation through the vascular system is a requirement for intravenous drug delivery systems such as nanoparticles. Vasculature connects the tissues, organs, and sites of pathologies in the body and provides an environment to introduce therapeutics into so that the therapeutics can reach their intended site. While it is well‐known that vascular features are dependent on their tissue/organ/pathology location, bioengineered systems that recapitulate essential features of our vascular systems have yet to be widely employed despite their widespread use as preclinical models for evaluation of many therapeutic modalities, nanoparticles included. In this issue of Bioengineering & Translational Medicine, a team led by Professor Hasan Abaci in the Department of Dermatology at Columbia University Irving Medical Center, discusses the evidence of microvascular heterogeneity between different organs, highlights the most recent 3D organ models, and shares their perspective as to how these recent systems recapitulate tissue‐specific features of the relevant microvasculature. This review is timely, as significant advances in developing organs‐on‐chips have led to their use in evaluating and predicting therapeutic responses and side effects and in capturing and describing biological phenomena. As these existing systems increase in complexity toward mimicking our tissues/pathologies better or even as individual chips are combined to mimic interactions and phenomena that occur during transport between organs, systems that recapitulate the heterogeneous and tissue‐specific characteristics of the vasculature may be a new tool to study an important delivery consideration.

DOI: 10.1002/btm2.10139

## IMPROVING NUCLEAR DELIVERY OF NANOPARTICLES

The current cutoff for diffusion into the nucleus of cells is reported to be 40 kDa, which limits the delivery of nanoparticles to the nucleus. Approaches to raising this cutoff could lead to better nanoparticle transport and delivery of drugs to the nucleus of cells. Researchers from the lab of Professor Victor Shahin from the Institute of Physiology II at the University of Münster, describe the use of 1,6‐hexanediol as a nuclear pore complex barrier breaker (NBB). Unlike many other described NBB, 1,6‐hexanediol has a history of safety through various exposure routes which was confirmed in this study as 1,6‐hexanediol exhibited insignificant toxicity in EA.hy926 cells. Importantly, it was demonstrated that 1,6‐hexanediol enabled the nuclear uptake of 150 kDa pDNA which could eventually have implications in the nuclear delivery of nanomedicines. While many efforts focus on circulation, biodistribution, and targeting of nanomedicines, new approaches to ensure and improve nanomedicine delivery into specific cellular compartments are needed. In this work, a new strategy to improve deliver larger entities to the nucleus could be leveraged in the future to improve nuclear delivery of nanomedicines for enhancing therapies based on gene editing or even chemotherapeutics.

DOI: 10.1002/btm2.10136

